# Using a Negative Binomial Regression Model for Early Warning at the Start of a Hand Foot Mouth Disease Epidemic in Dalian, Liaoning Province, China

**DOI:** 10.1371/journal.pone.0157815

**Published:** 2016-06-27

**Authors:** Qingyu An, Jun Wu, Xuesong Fan, Liyang Pan, Wei Sun

**Affiliations:** Dalian Center for Disease Control and Prevention, Liaoning Province, PR China; Institut Pasteur, FRANCE

## Abstract

**Background:**

The hand foot and mouth disease (HFMD) is a human syndrome caused by intestinal viruses like that coxsackie A virus 16, enterovirus 71 and easily developed into outbreak in kindergarten and school. Scientifically and accurately early detection of the start time of HFMD epidemic is a key principle in planning of control measures and minimizing the impact of HFMD. The objective of this study was to establish a reliable early detection model for start timing of hand foot mouth disease epidemic in Dalian and to evaluate the performance of model by analyzing the sensitivity in detectability.

**Methods:**

The negative binomial regression model was used to estimate the weekly baseline case number of HFMD and identified the optimal alerting threshold between tested difference threshold values during the epidemic and non-epidemic year. Circular distribution method was used to calculate the gold standard of start timing of HFMD epidemic.

**Results:**

From 2009 to 2014, a total of 62022 HFMD cases were reported (36879 males and 25143 females) in Dalian, Liaoning Province, China, including 15 fatal cases. The median age of the patients was 3 years. The incidence rate of epidemic year ranged from 137.54 per 100,000 population to 231.44 per 100,000population, the incidence rate of non-epidemic year was lower than 112 per 100,000 population. The negative binomial regression model with AIC value 147.28 was finally selected to construct the baseline level. The threshold value was 100 for the epidemic year and 50 for the non- epidemic year had the highest sensitivity(100%) both in retrospective and prospective early warning and the detection time-consuming was 2 weeks before the actual starting of HFMD epidemic.

**Conclusions:**

The negative binomial regression model could early warning the start of a HFMD epidemic with good sensitivity and appropriate detection time in Dalian.

## Introduction

The hand foot and mouth disease (HFMD) is a human syndrome caused by intestinal viruses like that coxsackie A virus 16, enterovirus 71 in infants and children[1–2], infectious strongly and easily developed into outbreak in kindergarten and school. In china, there was a sharp rise in incidence since the Chinese Ministry of Health (MOH) has listed HFMD as a notifiable Class-C communicable disease on May 2, 2008[3]and became one of the major infectious diseases affect children's health. According to the reference [3], 7,200,092 probable HFMD cases were reported to the China CDC surveillance system during 2008–2012, of which 3.7% were laboratory–confirmed and 0.03%died. The scientifically and accurately early detection of the start time of HFMD epidemic through estimating the baseline case number of HFMD is a key principle in planning of control measures and minimizing the impact of HFMD.

The Serfling regression has been extensively used to estimate a baseline describing the expected pattern of the historical disease in non-epidemic periods, for example, tuberculosis and influenza [4–5]. The model characterized the historical sequence of the disease time series by combination of a linear term with a trigonometric function describing the seasonal trend. Reference 4 presented the Serfling regression model in which the annual number of deaths attributable to influenza was calculated by summing the weekly excess over a period that only included the excluded weeks. Reference 5developed two Hidden Markov Models and selected the best model which considering the Serfling model results as reference. In this study, since it will produce too much negative data, so we didn’t use the Serfling regression to estimate a baseline case number of HFMD. Considering the data type of the HFMD cases was count data, we choice the Poisson regression or negative binomial regression model to estimate the baseline case number of HFMD. One of the reasons of that choice these two models was the Poisson regression is cited as a recommended approach for analyzing the count data. Another one was if the count data involve over-dispersion, the negative binomial regression was an alternative to analyze this kind of phenomenon.

In this study, we attempted to establish a reliable early detection model for start timing of hand foot mouth disease epidemic in Dalian using the Poisson regression or negative binomial regression model and to evaluate the performance of model by analyzing the sensitivity in detectability.

## Materials and Methods

### Study area

Dalian is the main coastal city of Liaoning Province, China and a major tourist city located at 38°43′-40°10′N latitude and 120°58′-123°31′E longitude. It had a population of 6.69 million in May 2011. Dalian has a warm continental monsoon climate and is in a marine temperate zone. The average annual temperature is 10.5°C with a maximum of 37.8°C, and a minimum of -19.1°C. The average rainfall is 550–950 mm and the total hour of annual sunshine is 2500–2800 hours [6].

### Data collection

The HFMD has been a notifiable communicable disease in China since May, 2008. The clinicians are required to report HFMD cases through the China information system for disease control and prevention within 24 hours. Weekly and Monthly HFMD cases in Dalian during the period of 2009 to 2014 were obtained from above information system. The HFMD cases included clinical and laboratory diagnosed cases. A clinical diagnosed HFMD case was defined as a patient with papular or vesicular rash on hands, feet, mouth or buttocks, with or without fever. A laboratory diagnose case was defined as a clinical diagnose case with laboratory evidence of enterovirus infection detected by reverse-transcriptase polymerase chain reaction (RT-PCR), real-time RT-PCR, or by virus isolation [7].

### Data analysis

According to the data fit for the basic Poisson or not, we use the Poisson regression or negative binomial regression model to estimate the baseline case number of HFMD. The assumption of the Poisson models is that the variance equal to mean, but due to unobserved heterogeneity and clustering, the HFMD data often display over-dispersion. The all variance of weekly cases of HFMD exceeds the mean of that in Dalian from 2009 to 2014 (Table 1). So finally we chose the negative binomial regression model to estimate the baseline case number of HFMD.

**Table 1:** The descriptive statistics of weekly numbers of HFMD cases in Dalian from 2009 to 2014.

Years	Mean weekly number of case	Variance weekly number of case
2009	156.85	32512.48
2010	260.94	125254.50
2011	111.88	20300.30
2012	300.62	229150.80
2013	213.67	111502.70
2014	145.75	37338.98
overall	198.15	95433.43

1.Establishing a negative binomial regression model to estimate the baseline: To estimate the weekly baseline case number of HFMD, we used the negative binomial regression model and calculated iteratively. The model was as follows:

$$Y_{t}=\beta_{0}+\beta_{1}t+\beta_{2}t^{2}+\beta_{3}\text{sin}\left(\frac{2\pi t}{52}\right)+\beta_{4}\text{cos}\left(\frac{2\pi t}{52}\right)+\varepsilon_{t}$$ (1)

Where *Y*_*t*_ is the number of HFMD cases reported in week t; *β*_0_, *β*_1_, *β*_2_, *β*_3_, *β*_4_ are the regression coefficients to be estimated; *ε*_*t*_ is a normally distributed error term. In this model *β*_0_, *β*_1_*t* and *β*_2_*t*^2^ represent the secular trend, $\beta_{3}\left(\frac{2\pi t}{52}\right)$ and $\beta_{4}\left(\frac{2\pi t}{52}\right)$ represent the seasonal trend. The Akaike Information Criterion (AIC) is used as the measure for goodness of fit in the model.

In this study, we reference the calculation steps of an adjusted Serfling regression model [8]. Firstly, we established a negative binomial regression model based on the actual weekly observed HFMD counts. Then, we excluded the actual observations which exceeded the fitted values from the first round of regression. And then, we established a negative binomial regression model again using the above cleaned data. We repeated this process until the AIC value stopped to increase. SPSS version 11.5 (SPSS, Chicago, IL) was used for data analysis. A *p*-value < 0.05 was considered statistically significant.

2. Determining the optimal threshold values: In this study, we tested difference threshold values and chosen the optimal alerting threshold between them during the epidemic and non-epidemic year. The epidemic year was defined together with the consultation result with local epidemiologists, consideration of the epidemiological characteristics of HFMD, namely the HFMD epidemics have been shown to occur in 2- to 3-year cycles [9] and all the weekly number of cases was over 200 during the epidemic peak zone. The gold standard of start timing of HFMD epidemic was calculated using circular distribution method. Specifically, we use the circular distribution methods to calculate the central tendency of the peak time points(r) and the peak time zone ($\bar{\alpha }\pm s$) of HFMD in Dalian from 2009 to 2014 as the following formula, and defined the start of peak time zone as the start timing of HFMD epidemic.

$$r=\sqrt{X^{2}+Y^{2}}$$ (2)

$$X=\left(\sum f_{i}\cdot \text{cos}\alpha_{i}\right)/\sum f_{i}$$ (3)

$$Y=\left(\sum f_{i}\cdot \text{sin}\alpha_{i}\right)/\sum f_{i}$$ (4)

$$\text{cos}\bar{\alpha }=X/r$$ (5)

$$\text{sin}\bar{\alpha }=Y/r$$ (6)

$$s=\frac{180{}^{\circ}}{\pi }\times \sqrt{-2\text{ln}r}$$ (7)

Where *α*_*i*_ (*i* = 1,2,⋯12) represents the angle of each month; *α* represents the average angle. Count 365 days a year and 360° all year round, so one day equal to 0.9863°.For example, calculated the average angle of January, because there are 31 days in January, the class mid-value of January is 15.5 days, so the angle of January is 0.9863 × 15.5 = 15.28765. By that analogy, there are 28 days in February, the accumulative days of class mid-value of February is 31 + 14 = 45 days, so the angle of February is 0.9863 × 45 = 44.3835 [10]. *f*_*i*_ is the month’s number of HFMD cases. DPS(Data Processing System) was used for data analysis. A *p*-value < 0.05 was considered statistically significant.

### Ethics statement

This study was approved by the ethics committee of Dalian center for control and prevention. Dalian HFMD data during 2009 to 2014 were obtained from the China information system for disease control and prevention. No informed consent was required because there were no ethical issues relevant to the study design and no individual-level analysis was performed. The information contained in the patient’s records was anonymized and de-identified prior to analysis. The data mainly date of onset were aggregated and analyzed.

## Results

### Descriptive analysis

From2009 to 2014, 62022cases of HFMD were reported (36879 males and 25143 females) in Dalian, Liaoning Province, China, including 15 fatal cases (Table 2). The median age of the patients was 3 years (range: 1days to 75 years).

**Table 2:** The numbers of reported HFMD cases in Dalian from 2009 to 2014.

Years	Number of cases of HFMD			Incidence rate(/100,000)
	clinical diagnose case	laboratory diagnose case	overall	
2009	8258	55	8313	137.54
2010	13273	296	13569	224.48
2011	5751	67	5818	86.92
2012	15185	447	15632	231.44
2013	10580	531	11111	165.33
2014	7408	171	7579	111.29
overall	60455	1567	62022	159.50

### Circular statistical analysis

**Fig 1:**
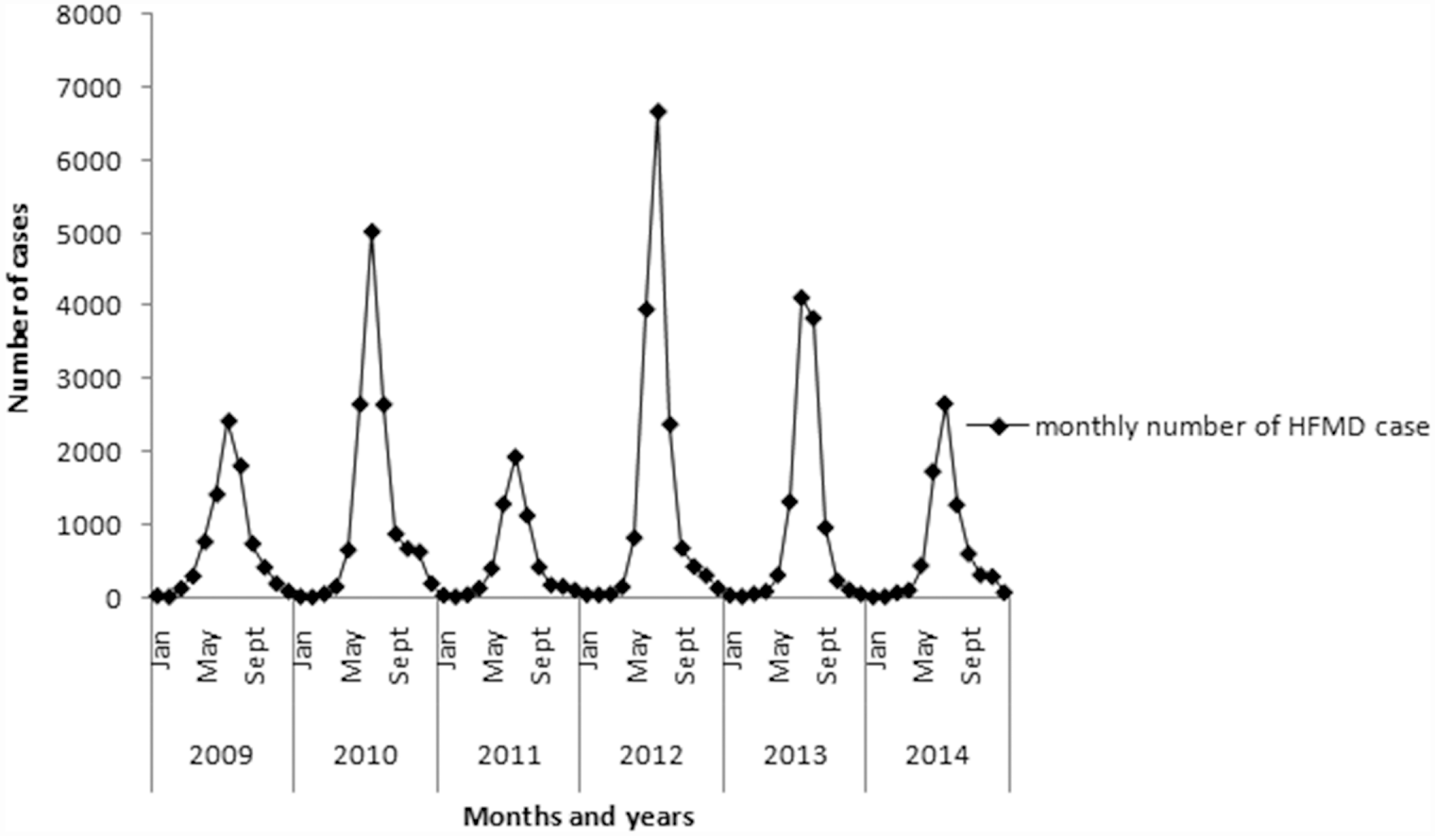
Monthly cases of HFMD in Dalian 2009–2014.

The monthly cases of HFMD reported from Dalian during 2009–2014 are shown in Fig 1. Although HFMD cases occurred throughout the year, June, July and August each year were the months with the highest incidence of HFMD. We used the circular distribution test to calculate the peak day and the peak period of HFMD for each year. The occurrence of HFMD from 2009 to 2014 in Dalian was concentrated, namely there was central tendency (overall r = 0.76, P<0.01), the concentrated vector quantity r ranged from 0.692 to 0.842. The peak day fluctuated between July 14th to 29th and the peak period of incidence ranged from May 31st to September 8th during 2009-2014(Table 3).

**Table 3:** The time analysis result of HFMD in Dalian 2009–2014.

Years	r	P	peak time point	peak time zone
2009	0.69	P<0.01	July 20th	May 31st-September 8th(22nd week-37th week)
2010	0.74	P<0.01	July 24th	June 8th-September 8th(23rd week-36th week)
2011	0.73	P<0.01	July 18th	May 31st-September 3rd(22nd week-35th week)
2012	0.81	P<0.01	July 14th	June 6th-August 21st(23rd week-34thweek)
2013	0.84	P<0.01	July 29th	June 24th-September 2nd(26th week-36th week)
2014	0.74	P<0.01	July 20th	June 4th-September 4th (23rd week-36th week)
overall	0.76	P<0.01	July 21st	June 7th-September 3rd

All the weekly number of cases was over 200 during the epidemic peak zone in Dalian from 2009 (16/16) to 2010(14/14) and from 2012(12/12) to 2013(11/11). There are 21.43% (3/14) and 14.29% (2/14) of the weeks which the number of cases was less than 200 in 2011 and 2014 respectively. In this study take into consideration 3 aspects, including consultation result with local epidemiologists, the epidemiological characteristics of HFMD and the weekly number of cases criteria during the epidemic peak zone, the epidemic year was defined as the period from2009 to 2010 and from 2012 to 2013, during which the incidence rate ranged from 137.54 per 100,000 population to 231.44 per 100,000population; the other years were defined as the non-epidemic year, the incidence rate in 2011 and 2014 was 86.92 per 100,000 population and 111.29 per 100,000 population respectively(Table 2).

### The negative binomial regression model fitting

Since the negative binomial regression model usually require historical data over several years, we established the model using the weekly incidence of HFMD from 2009 to 2012 in Dalian to estimate the baseline data. After baseline data construction, a simulation was conducted for the year 2013 and 2014.

In the first run, we established a negative binomial regression model based on the actual weekly observed HFMD counts. The AIC value was 2257.51. After, we excluded the actual observations which exceeded the fitted values from the first round of regression, the AIC value decreased to1355.53.We continued to carry out multiple rounds of iterative regressions, the AIC value decreased to 147.28 and then decreased to 52.45, but there was no statistical significance for the parameter (Table 4).So we selected the model with the AIC value 147.28 to construct the baseline level(S1 Database) and the equation for the model was:$Y_{t}=\text{−}13.12\text{−}1.57\text{ sin}\left(\frac{2\pi t}{52}\right)\text{−}2.16\text{ cos}\left(\frac{2\pi t}{52}\right)$.

**Table 4:** The Akaike Information Criterion (AIC) values and the parameters estimates for different rounds of iterative regression.

Round time	AIC	Parameter estimates				
			Estimates	Std Error	Wald Chi-Square	p-value
Round 1	2257.51	Constant	-11.60	0.32	1348.77	0.00
		t	0.02	0.01	1.88	0.17
		t^2^	0.00	0.00	2.55	0.11
		t^3^	<0.001	<0.001	2.99	0.08
		cos1^Δ^	-1.75	0.09	364.36	0.00
		sin 1^※^	-1.32	0.13	109.76	0.00
Round 2	1355.53	Constant	-11.86	0.12	10559.54	0.00
		cos1^Δ^	-1.88	0.13	197.63	0.00
		sin 1^※^	-1.39	0.12	133.30	0.00
Round 3	667.72	Constant	-12.23	0.18	4651.08	0.00
		cos1^Δ^	-1.99	0.24	71.54	0.00
		sin 1^※^	-1.37	0.15	79.13	0.00
Round 4	274.79	Constant	-12.69	0.33	1452.35	0.00
		cos1^Δ^	-2.09	0.42	24.88	0.00
		sin 1^※^	-1.35	0.29	21.43	0.00
Round 5	147.28	Constant	-13.12	0.61	468.47	0.00
		cos1^Δ^	-2.16	1.14	3.91	0.05
		sin 1^※^	-1.57	0.54	8.5	0.00
Round 6	52.45	Constant	-13.28	2.97	19.96	0.00
		cos1^Δ^	-1.64	3.13	0.27	0.60
		sin 1^※^	-1.96	1.87	1.11	0.29

^Δ^:$\text{cos}1=\text{cos}\left(\frac{2\pi t}{52}\right)$,

^※^:$\text{sin}1=\text{sin}\left(\frac{2\pi t}{52}\right)$

### Determining the optimal threshold values

When the number of observed cases from 2009 to 2012 exceeded the baseline data a certain value (threshold value) two successive, an alert would be generated, indicating the starting of HFMD epidemic. So, firstly we calculated the difference between the observed value and the baseline data. Then, we test 9 candidate threshold values from 100 to 500, the interval is 50 for the epidemic year, and the half value of them for the non-epidemic year. And then, we calculated their sensitivity to determine the optimal threshold. We defined the early warning succeed as the detection time (the median number of weeks from the start of peak time zone to the first alert) larger than one week. If the first alert generated on the same week that start of peak time zone, early warning was failure.

**Fig 2:**
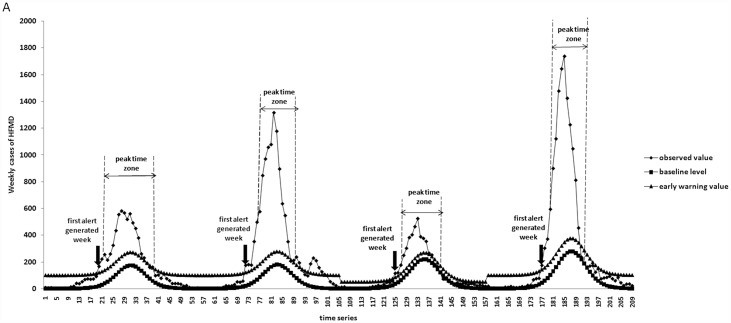
**The different early warning value and the early warning result for HFMD in Dalian** (A)threshold value was 100 for the epidemic year, 50 for the non-epidemic year;(B)) threshold value was 150for the epidemic year, 75 for the non-epidemic year;(C) threshold value was 200 for the epidemic year, 100 for the non-epidemic year; (D) threshold value was 250 for the epidemic year, 125 for the non-epidemic year; (E) threshold value was 300 for the epidemic year, 150 for the non-epidemic year; (F) threshold value was 350 for the epidemic year, 175for the non-epidemic year; (G) threshold value was 400 for the epidemic year, 200for the non-epidemic year; (H) threshold value was 450 for the epidemic year, 225for the non-epidemic year; (I) threshold value was 500 for the epidemic year, 250for the non-epidemic year; early warning value was the sum of baseline value and the different threshold value.

Fig 2 and Table 5 show the different threshold value and sensitivity separately for the epidemic year and non-epidemic year. The early warning value was the sum of baseline value and the different threshold value in Fig 2. The threshold value was 100 for the epidemic year and 50 for the non- epidemic year had the highest sensitivity (100%) and the detection time-consuming was 2 weeks before the actual starting of HFMD epidemic.

**Table 5:** The different threshold value and sensitivity for early warning at the starting of HFMD in Dalian.

threshold value^Δ^	week of first alert generation from 2009 to 2012				sensitivity (%)
	2009	2010	2011	2012	
100/50	20th	19th	20th	20th	100
150/75	21st	19th	22nd	21st	75
200/100	25th	22nd	22nd	21st	50
250/125	26th	22nd	23rd	22nd	50
300/150	26th	22nd	23rd	23rd	25
350/175	27th	23rd	24th	23rd	0
400/200	27th	23rd	25th	23rd	0
450/225	-^※^	24th	25th	24th	0
500/250	-^※^	25th	27th	24th	0

^Δ^:a/b, a for the epidemic year, b for the non-epidemic year;

^※^:there was no week to detect

### Prospective early warning

**Fig 3:**
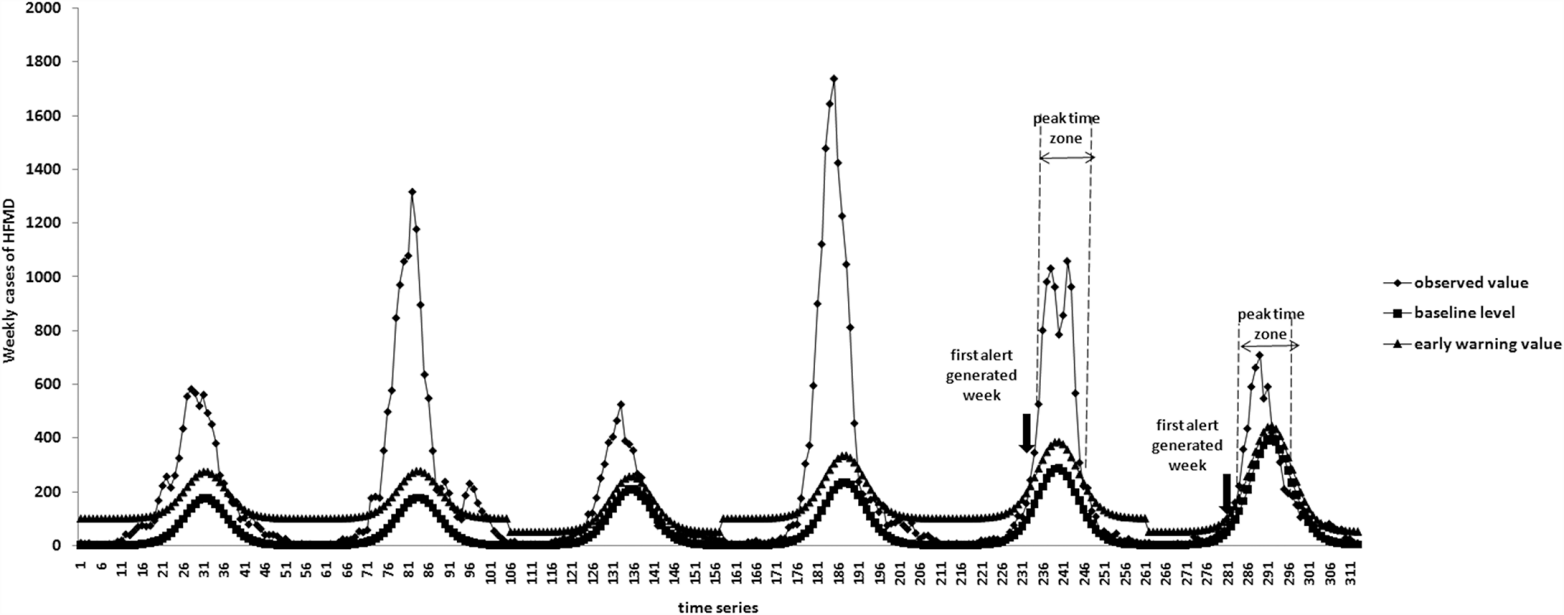
The early warning result for HFMD in Dalian during 2013–2014 using the number of predicted baseline data obtained from the negative binomial regression.

Fig 3 show the number of predicted baseline data obtained from the negative binomial regression for 2013 and 2014. The year of 2013 was an epidemic year, so the threshold value was 100. According to above criteria, the week of first alert generated was the 25th week. In the same way, the year of 2014 was a non-epidemic year, so the threshold value was 50 and the week of first alert generated was the 21st week. The start of peak time zone 26th week and 23rd week in 2013 and 2014 were then observed. It showed that the negative binomial regression model could early warning the start of HFMD epidemic 1.5 weeks before the actual start of peak time zone.

## Discussion

In recent years, HFMD epidemics have been common in East and Southeast Asia since the first reported in 1957[11].From January 2009 to December 2014, 62022 HFMD cases were reported in Dalian, the average incidence rate was 159.50 per 100,000 populations. The average incidence rate was higher than the reported average incidence levels of other city in Liaoning provinces and lower than the part of southern province in China [12–16].

The peak time zone was from May 31st to September 8th, with the peak time point fluctuated at from July 14th to 29th. This result was consistent with a previous study in China, which conclusion that the incidence peak of HFMD in northern china was observed in summer [17]. This is likely associated with the easy propagation of virus in the seasons of high temperature and high humidity [18].In this study, the circular distribution test was used to analyze the seasonality of HFMD. Compared with using the constituent ratio or relative ratio, the circular distribution method could exactly provide accurate peak time and peak phase for the seasonal disease [19], and the gold standard of start timing of HFMD epidemic can be defined accurately and objectively. This was a strength of our study.

The another strength of our study was different optimal threshold value for the different year. The number of weekly HFMD cases differed greatly between the epidemic year and the non-epidemic year, especially during 2010 and 2012 to 2013, the mean weekly number of cases were over 200, thus if we used the same threshold value during the epidemic and non-epidemic year, the false alarm rate must be increased or the lead-timing too more to rational allocation of public health resources.

In this study, we aimed to establish a new reliable early detection model for start timing of hand foot mouth disease epidemic in Dalian and to evaluate the performance of model. Our study result indicated that the negative binomial regression model had good sensitivity (100%) in the detection of start timing to HFMD and could to detect2.5weeks for the epidemic year(2009–2010,2012–2013), 2 weeks for the non-epidemic year(2011,2014) before the actual starting of HFMD epidemic. After the initial infection, the host remains in a latent stage for a period of time before becoming infectious [20]. For HFMD, the incubation period of HFMD was 3 to 10 days[21],so 2 to 2.5 weeks leading time were enough to implement the prevention and control measures, such as promotion of health education, case isolation, disinfection of affected setting and so on [1], which can decrease the morbidity during the upcoming epidemic season. Because of the epidemic started, there would be a sharp increase in the number of HFMD cases and then reached the highest incidence soon.

This study had several limitations. First, we used only one disease with high incidence rate in Dalian as object of study, so if we used the other diseases with relatively low incidence rate, the above results will not generalize or the threshold value should readjust. Second, the definition of the epidemic and non-epidemic year was empirical, especially the incidence rate of 2009 and 2014 was not statistically difference, but they were defined by the epidemic year and non-epidemic year respectively.

## Conclusions

With further studies, the negative binomial regression model might be useful for early warning at the starting of other seasonal infectious diseases epidemic and the results could furnish a scientific reference for policy makers and health agencies.

## Supporting Information

S1 Database Database containing weekly time-series of HFMD and the baseline value obtained from the negative binomial regression during 2009–2012 in Dalian. (XLSX)
